# Seeking Something Beyond Themselves: A Concept Analysis of Spiritual Awakening Experiences at the End of Life

**DOI:** 10.3390/nursrep15100358

**Published:** 2025-10-08

**Authors:** Manuela Monteiro, Joel Vitorino, Marina G. Salvetti, Carlos Laranjeira

**Affiliations:** 1 School of Health Sciences, Polytechnic University of Leiria, Campus 2, Morro do Lena, Alto do Vieiro, Apartado 4137, 2411-901 Leiria, Portugal; 2 Centre for Innovative Care and Health Technology (ciTechCare), Polytechnic University of Leiria, Campus 5, Rua das Olhalvas, 2414-016 Leiria, Portugal; 3 Medical-Surgical Nursing Department, School of Nursing, University of São Paulo, São Paulo 05403-000, Brazil; 4 Comprehensive Health Research Centre (CHRC), University of Évora, 7000-801 Évora, Portugal

**Keywords:** spiritual awakening, spiritual awareness, consciousness, end-of-life, concept analysis, palliative care, nursing

## Abstract

**Background/Objectives:** End-of-life (EoL) experiences are critically important for everyone involved, giving rise to a set of needs that extend far beyond bio-physiological aspects, to encompass the spiritual dimension as the core of human beings. Understanding the processes of spiritual awakening (SA) assists palliative care professionals in enhancing the quality of care provided to individuals with life-threatening illnesses, as well as to their families. SA is a fundamental occurrence linked to the fulfilment of our spiritual needs when facing an existential crisis, such as the proximity of death. However, its conceptual boundaries need to be clarified to provide qualified and humanized palliative care. Therefore, this study aims to identify the key attributes, antecedents, consequents, and empirical referents of SA at EoL, as well as to clarify the concept’s existing ambiguities. **Methods:** Walker and Avant’s eight-step concept analysis was used. A literature search was conducted in May 2025 across three databases (PubMed, CINAHL and Scopus). **Results:** Following the review, 21 articles were included for analysis. The concept analysis revealed four main attribute domains: (1) sensory–perceptual domain; (2) affective/cognitive domain; (3) relational domain; and (4) transcendental domain. Moreover, spiritual consciousness and the existential matrix were antecedents to this concept; revaluation of beliefs, finding spiritual serenity and inner freedom, fostering spiritual growth, and the desire to leave a legacy were its consequences. **Conclusions:** The concept of SA at the EoL reveals itself to be a complex and multifactorial phenomenon, with a profound impact on a person’s confrontation with finitude. Recognizing and integrating SA into palliative care allows for a more comprehensive understanding of human consciousness. To deal with SA experiences in healthcare settings, a multifaceted approach is needed. This encompasses acknowledging spirituality as a determinant of health, including spiritual care in standard practice, and offering education and training on spiritual care competence for healthcare practitioners. Further transdisciplinary research should be undertaken to explore SA phenomenological variations, guide clinical interventions, and evaluate SA impacts on spiritual well-being and spiritual growth.

## 1. Introduction

Recently, there has been heightened emphasis on helping professionals enhance well-being by addressing mental, bodily, and emotional dimensions within the framework of human spiritual requirements [1,2]. Spiritual needs pertain to our biopsychosocial-spiritual whole, our need to seek and discover meaning, purpose, and worth in our lives [3,4]. Various religions and spiritual practices throughout history and worldwide have sought to provide frameworks to assist individuals in meeting their spiritual requirements.

According to the Modified Biopsychosocial-Spiritual Model, spirituality constitutes the core dimension of integral human development, and together with the outer layers (physical, psychological, and social levels) constitutes a whole person [5]. Spirituality is a distinctive facet of human experience, varying for each individual, and may gain significance when confronting life-threatening illness. The lived experiences of spirituality can differ greatly, as seen by our beliefs, values, rituals, traditions, and practices. Certain individuals may completely reject the concept of spirituality, preferring terms like connection, meaning, and purpose when reflecting on the existential aspects of life. In this context, we utilize the term spirituality in a broad and inclusive sense, acknowledging unique experiences of connection and meaning or purpose [6]. According to European Association of Palliative Care (EAPC), spirituality is the “dynamic dimension of human life that relates to the way persons (individual and community) experience, express and/or seek meaning, purpose and transcendence, and the way they connect to the moment, to self, to others, to nature, to the significant and/or the sacred” [7]. Carl Jung theorized that spirituality manifests through the psyche’s natural drive toward individuation [8]. He believed that “before one achieves spiritual individuation, one unavoidably ought to encounter darkness and descent” [8]. Therefore, to mitigate human suffering, “people need to face their own souls” [8], which means activating characteristics of universal spiritual experience, such as love and altruism, spiritual reflection and contemplative practice [9].

Spirituality is related to the concept of spiritual awakening (SA), with the latter often described as a call to a significant shift in awareness and understanding of oneself and the world [10]. SA is a fundamental occurrence linked to the fulfilment of our spiritual needs. Despite diverse traditions and practices with distinct frames and explanations of SA, it generally denotes a transformation in perception, cognition, and behavior [11,12]. This transformation leads to inner tranquility, receptiveness, and a feeling of oneness, as well as heightened sensitivity, generosity, genuineness, and awareness of the present moment [11]. SA is typically seen as a very uncommon mental state and is frequently linked to individuals involved in religious or spiritual practices [11]. Recent research in transpersonal psychology indicates that SA is a phenomenon linked not solely to individuals adhering to religious or spiritual traditions, but also to those devoid of any such affiliations [11,12,13]. Furthermore, SA is not merely an abrupt occurrence characterized by a drastic transformation in perception, cognition, and behavior; individuals, irrespective of their association with any specific religion or spiritual framework, may undergo SA progressively [11,12]. A Gallup Poll study reveals that approximately 41% of American adults experience SA; nonetheless, this phenomenon is private for most individuals and is rarely discussed publicly [14]. Evidence reveals that persons who experienced an intense spiritual experience were reluctant to disclose it to a healthcare professional, primarily due to fears of dismissal or being deemed pathological [10,15]. For instance, when participants were queried about sharing their experience with a healthcare professional, fewer than half responded affirmatively, and 80% of them expressed dissatisfaction with the caregiver’s response [10].

SA has been documented across diverse religions, spiritual traditions, cultures, and epochs, exhibiting consistent characteristics despite differing terminologies (e.g., Bodhi in Buddhism, Sahaj Samadhi in Hinduism, Buqa in Sufism, and Deification in Christianity) [15]. Academic discussions about SA indicate that people in peak experiences and kundalini awakenings experience temporary states of unitive consciousness, characterized by the dissolution of individual boundaries; a sensation of unity with others, nature and the universe; a sense of intensive happiness; a transcendence beyond time and space; and a connection with the divine [16,17]. These experiences contrast with a more sustained, stable state of “wakefulness” or liberation, sometimes referred to as a permanent awakening or enlightenment [18].

Taylor [11] writes that a “few people are born naturally awake, some awaken gradually”, but for most it happens suddenly, such as when people with life-limiting illnesses realize their imminent death. The proximity of death can instigate a profound inner transformation, an inward upheaval that significantly diverges from previous experience [16]. According to Cobo-Medina [19], the phenomenon termed “personal uninstall” influences all aspects of a person. During these moments, the dying person experiences what is referred to as the “ultimate existential crisis” and goes through a period of emptiness, suffering and inner turmoil [17]. It is perhaps unsurprising that SA acts as a buffer against stress and improves coping against the depressive effects of stressful events, promoting positivity, equanimity, optimism, peace, resilience and the recognition of one’s unique existence in the context of prevailing circumstances [20].

Providing spiritual care involves an ethical stance that focuses on an individual’s suffering and capability to find a sense of purpose, which implies merging a human-centered approach with an evidence-based practice [19,21,22,23]. Key components of spiritual care encompass presence, intentionality, and compassion [24]. Presence is the practice of engaging with patients in a manner that recognizes and respects their holistic essence, emphasizes the establishment of a real interpersonal environment, and treats patients as distinct individuals. Intentionality involves performing an action with empathy and a desire for maximum benefit. Compassion is recognizing a person’s feelings, seeking to understand their anguish, and taking action to alleviate that suffering [25,26,27]. Enhancing health professionals’ comprehension of people’s SA experiences requires further research and the incorporation of new frameworks for conceptualizing and examining diverse aspects of SA.

Though there have been several studies characterizing the nature of SA and its transformation among the general population [12,17], few studies have been conducted in people living with life-limiting illnesses [28,29]. Likewise, the concept of “spiritual awakening experiences at the EoL” lacks greater conceptual clarity to deepen the understanding of the repercussions of approaching death and its intersection with SA. Therefore, it is essential to incorporate a conceptual analysis of “spiritual awakening experiences at the EoL” to clarify the concept and promote its practical application in the provision of qualified and humanized care. To this end, the following research question was formulated: What evidence is available on the attributes, antecedents, consequences, and empirical referents of the concept “spiritual awakening experiences at the EoL”? Assuming that all humans are spiritual beings, the aims of this study were: (a) to define the attributes of the concept of “spiritual awakening at the EoL”; (b) to determine the antecedents and consequences of the concept; and, finally, (c) to identify its empirical referents.

## 2. Materials and Methods

### 2.1. Study

Walker and Avant’s [30] concept analysis method guided this study. In this eight-step interactive approach, the first step consists of selecting the concept to be analyzed, along with recognizing synonymous terms. In the second step, the study determines the aims or purpose of the analysis. The third and fourth steps identify the concept’s use in the literature and determine its defining attributes, enabling a comprehensive and detailed analysis. In the fifth and sixth steps, a model case and additional cases—a borderline case and a contrary case—are identified to support the discussion and comparative analysis of the attributes. The seventh step involves identifying the antecedents and consequences of the concept, where antecedents are the elements prior to the concept and consequences are the results of the concept’s occurrence. Finally, the eighth stage involves defining the empirical referents that allow the analysis of the concept under study [30].

### 2.2. Literature Search

A literature search was carried out in May 2025 using the following databases: Medline (PubMed), Cumulative Index to Nursing and Allied Health Literature (CINAHL) and Scopus. The search string was established using the Medline database and subsequently adapted to other databases. A combination of MeSH (Medical Subject Headings) and keywords employing Boolean operators and truncation (*) was utilized in the search strategy: ((“Spiritual awakening” [Title/Abstract] OR “spiritual outbreak” [Title/Abstract] OR “personal awakening” [Title/Abstract] OR “consciousness” [MeSH Terms] OR “consciousness” [Title/Abstract] OR “spirit*” [Title/Abstract] OR “Self-awareness” [Title/Abstract] OR “sacred experience” [Title/Abstract] OR “Transpersonal psychology” [Title/Abstract] OR “mystical experience” [Title/Abstract] OR “Salvation” [Title/Abstract] OR “Rebirth” [Title/Abstract] OR “Awakening soul” [Title/Abstract] OR “Enlightenment” [Title/Abstract] OR “Epiphany” [Title/Abstract] OR “Transcendence” [Title/Abstract] OR “Mind Self-knowledge” [Title/Abstract] OR “Revelation” [Title/Abstract] OR “Spiritual transformation” OR [Title/Abstract] “Self-consciousness” OR [Title/Abstract] “Spiritual awareness” [Title/Abstract] OR “Spiritual revival” [Title/Abstract] OR “positive disintegration” [Title/Abstract] OR “Inner force” [Title/Abstract]) AND (“palliative care” [MeSH Terms] OR “palliative care” [Title/Abstract] OR “end of life” [MeSH Terms] OR “end of life” [Title/Abstract] OR “Terminal care” [MeSH Terms] OR “Terminal care” [Title/Abstract] OR “Death” [MeSH Terms] OR “Death” [Title/Abstract] OR “Dying” [Title/Abstract]) AND ((ffrft [Filter]) AND (english [Filter] OR portuguese [Filter] OR spanish [Filter]) AND (alladult [Filter])). Filters: Free full text, English, Portuguese, Spanish, and all adults.

Included were primary (qualitative, quantitative, and mixed methods) and secondary studies (narrative, integrative and systematic reviews) involving theoretical and experiential analyses of SA in an EoL context, written in English, Portuguese, and Spanish (languages spoken by the authors), and with no time restrictions. Studies without peer review, those without full-text availability, and dissertations, theses, editorials, and conference proceedings were excluded. The reference lists of included studies were manually reviewed to identify additional studies missing in the database search.

### 2.3. Data Analysis

A data extraction tool (spreadsheet) was used to obtain title, author(s), year of publication, aims, setting/participants and main findings on “spiritual awakening experiences at the EoL” from the final sample of articles included in this concept analysis. Three researchers (M.M., J.V., and C.L.) blind screened, selected, evaluated, and extracted data from studies. Research team members discussed inconsistencies to establish consensus. A qualitative thematic analysis was performed on selected articles after reading, coding, and theming [31]. The data themes were identified by inductive coding and evaluated by a team of social and healthcare professionals with expertise in conducting research in the field of palliative care. Organization of the studies’ information allowed identification of the concept’s antecedents, attributes, consequences, and empirical referents. After meaning saturation was reached (where data stops revealing new insights), extracted data were classified and labelled.

## 3. Results

**Figure 1:**
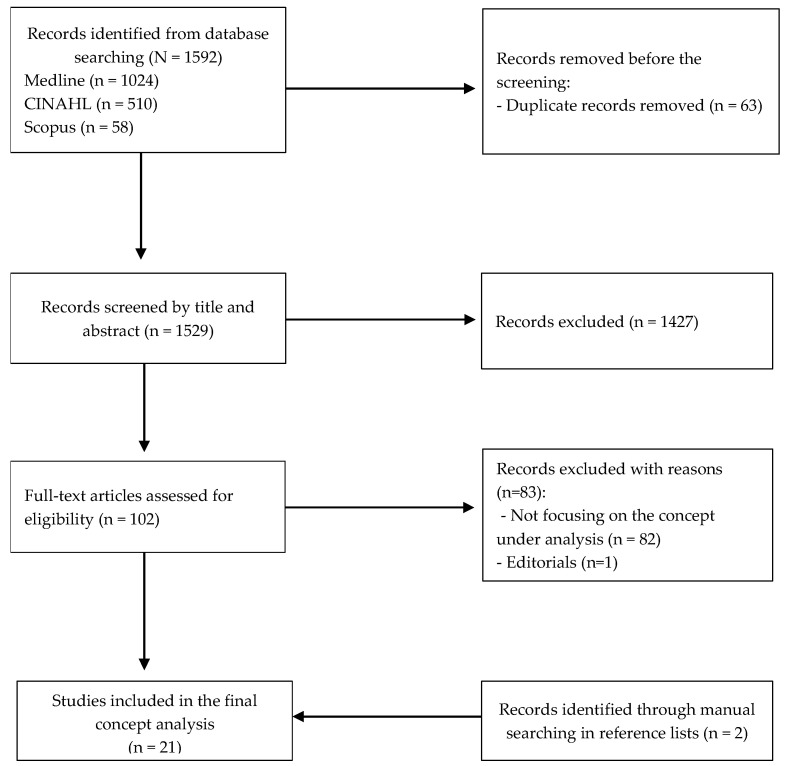
Systematic search strategy regarding concept analysis of SA experience at the EoL.

The initial database search yielded 1592 publications in total, of which 63 were eliminated as duplicates, leaving 1529 articles. After screening titles and abstracts, 1427 articles were excluded. Consequently, 102 papers were thoroughly examined to ascertain their eligibility. After 83 articles were removed, 19 articles remained in the final sample. By looking through the articles’ reference lists, eight more articles were found. In the end, 21 articles were included in the final sample (Figure 1). All documents were published between 2010 and 2024 and originated from the following countries: United States (N = 6), Canada (N = 2), United Kingdom (N = 3), China (N = 1), Switzerland (N = 1), Italy (N = 2), Brazil (N = 1), Turkey (N = 1), India (N = 1), Denmark (N = 1), Iceland (N = 1), and Germany (N = 1). Most studies were qualitative (N = 13), followed by quantitative studies (N = 4), mixed-method studies (N = 3), and finally one discussion paper. A summary of the included publications is shown in Supplementary Table S1—Characteristics of the studies included in the review (N = 21).

### 3.1. Use of Term in Dictionary Definitions

According to the Cambridge Dictionary [32], *spiritual awakening* is a shift in consciousness, a perception of reality that was previously unknown or misunderstood, revealing oneness with all existence. The central idea is that absolute truth transcends human beliefs and reveals an essential unity behind true nature. This energetic essence is timeless, infinite, and eternal. In turn, Dictionary Online [33] offers us a view of “a perception or inspiration about connection with an entity or entities beyond the immediate, physical world, with God or another intangible, sacred being.” The Collins Online Dictionary distinguishes both concepts, defining *awakening* as “a feeling of awareness or beginning” of something, while *spiritual* refers to “people’s thoughts and beliefs, rather than their bodies and physical environment” [34]. The *Merriam-Webster Dictionary* [35] also clearly distinguishes the concepts of *awakening* and *spiritual*. It defines *awakening* as a transformative “realization” or “coming into awareness,” focused on self-perception or understanding of the surrounding reality [35]. In contrast, *spiritual* is described as pertaining to the “spirit” and the “incorporeal,” often associated with “sacred matters, religious values, faith, and inner search” [35]. This term may carry an ecclesiastical (rather than secular) connotation or express spiritual affinities between individuals or places. Among its derived forms is *spiritually* (adverb), which denotes a spiritual expression marked by inner depth or connection to the sacred.

### 3.2. Defining Attributes

According to Walker and Avant [30], identifying and analyzing attributes is a fundamental step in clarifying and operationalizing a concept. SA at the EoL is a complex experience that involves multiple dimensions of being. For the purpose of analysis, the attributes were organized into four interdependent domains (Table 1): (1) sensory–perceptual domain; (2) affective/cognitive domain; (3) relational domain; and (4) transcendental domain. Each domain will be explored below, along with the corresponding categorization of attributes.

#### 3.2.1. Sensory–Perceptual Domain

SA often involves changes in temporal–spatial perceptions, which are closely linked to self-awareness [37]. It manifests as intense bodily sensations, often described as sensations of heat or energy flows, electrical sensations in the extremities, disturbances in the digestive system, and spontaneous involuntary movements, including tremors, tingling, and pins and needles in the body [38]. These manifestations permeate the body [39] and are closely linked to internal energetic experiences perceived as transformative in the perception of oneself and the surrounding world [36,38,39].

Energetic hypersensitivity and the modification of the perception of time and space lead the person to recognize an identity between the individual self and universal consciousness. The practices of tantra and yoga, as well as contemplative practices, such as mindfulness and meditation, act as catalysts for states of expanded consciousness [38,41,42].

#### 3.2.2. Affective/Cognitive Domain

SA frequently involves an affective/cognitive dimension characterized by a sense of morality and awareness of the present moment, by an expansion of consciousness, capable of identifying negative thoughts, fears and anxieties, thus allowing an amplified spiritual connection and universal outlook [38,40,41]. A sense of morality reminds people that spirituality is not an escape from the hurly-burly of the real world. Rather, SA is the means to remain in the world and not be overwhelmed by it. In parallel, it includes the development and consolidation of a positive affect [43,44,45] that manifests itself in the experience of the here and now [45,46]. This can be intensified through unifying silence, which connects the individual to the authentic self and their essence [36,42,43,44,45,46]. Several practices facilitate unifying silence, such as prayer (which provides peace of mind and well-being) and other spiritual practices, including candlelight meditation, gratitude and mindfulness [42]. Also, the use of palliative sedation or the use of psychoactive substances, such as psilocybin [50], can also produce changes in emotional regulation and cognitive reappraisal of spiritual self-awareness [38,39,46]. However, it should be noted that “there is no medication for alienation, loneliness, despair, meaninglessness, and fear of death. When a person faces death, what is most important is that they still find meaning in life through love, faith, hope, and a sense of completion” [47]. This experience is associated with the expansion of moral, affective, and identity consciousness, which deepens the feeling of connection with humanity [36,38,40,46,47].

#### 3.2.3. Relational Domain

In the relational dimension, SA in EoL leads to the dissolution of bonds (e.g., if a friendship was built on mutual venting, and a person no longer wants to complain, the bond may dissolve), psychological and social detachment, nonmaterialism, and the development of altruism, compassion, empathy, and forgiveness [36,43,45,47]. This phenomenon is described as “opening of heart” and displays a flow of love, kindness, and compassion for the self, others, and the whole. The central desire to give and receive love emerges, fostering integrated, compassionate, and intimate states of consciousness, in “one web of life that connects every exquisite being” and transcends cultural and religious barriers [38,43,45,48,49]. SA can surpass traditional religious structures, leading to a more personal, ecumenical, and existential spiritual experience. People often report a conscious distancing from formal religious institutions (“Turning away from the church without losing faith; creation of personal spiritual values” [38]), which indicates a dissociation between spirituality and conventional religious group identity. This transformation does not necessarily represent a loss of faith, but rather a reformulation of its contours, based on one’s own spiritual values, often resulting in more eclectic forms of religious viewpoint. Therefore, the spirit deserves recognition along with mind and body as an element of human formation [48].

#### 3.2.4. Transcendental Domain

In the transcendental domain, SA in EoL is characterized by the dissolution of the ego, which can trigger a feeling of “energy flooding” and lead to a state of expanded consciousness, with neurobiological changes and experiences of fusion with a supreme reality, such as God or the universe [38,39,45]. The transition of consciousness from an egocentric state (pre-transition) [defined by rational reasoning, conceptual understanding, introspection, impulse regulation, willpower, organizational skills, decision-making and planning] [36] to a state beyond the ego (post-transition), of deep existential integration, which occurs mainly in moments of serenity and emotional stability [36,38,39,40]. This existential integration highlights the importance of seeking something beyond oneself [42], especially in moments of vulnerability [42,43,48]. This allows individuals to feel heard and respected in their life as a journey [51], to find meaning [48], and express the desire to leave a legacy [42], especially through their family connections, which reinforces the symbolic continuity of life itself. The search for inner peace and a sense of belonging to a higher and transcendent reality offers faith and hope in the afterlife [36,43,47,52,53]. Thus, faith constitutes a transformative vital force, facilitating the serene acceptance of death and reinforcing the connection to a transcendent reality, making sense of the past and transforming negative perspectives into positive ones [13,42,43,44,47,51,52,53].

### 3.3. Case Examples

A case model, a contrary case, and a borderline case—all taken from real-life experiences of palliative care patients—will be provided to elucidate the defining attributes of the concept under examination [30]. Since no personal information was used and all names attributed to the characters are fictional, ethical approval was not required.

#### 3.3.1. Case Model

The model case is an ideal example of the concept in practice, showcasing all its unique attributes [30]. Virpi, a 33-year-old Hindu woman, originally from India and currently residing in an urban area in central mainland Portugal, is married with two children. Since 2024, she has been working as a healthcare assistant at a private medical institution. From an early age, she sought answers that would provide meaning to her existence. Unlike most, she never identified with standardized trends or conventional ways of living, and she began practicing meditation at a young age as a means of connecting more deeply with herself. In December 2023, she was diagnosed with stage III breast cancer, a moment that marked a turning point in her personal journey. After oncotherapy and radiotherapy treatments, she began seeking new ways to find meaning in her life, developing a profound sense of detachment from material possessions, becoming more interested in being, both for herself and for others, rather than having. She aimed to transform her way of living, becoming a volunteer for a non-governmental organization (NGO) that supports homeless people, thus complementing her professional role with what she considers to be her purpose in the here and now: to help others achieve their personal or professional goals and reach their full potential. During her meditative practices, Virpi has described experiencing new bodily sensations, which she links to energetic and vibrant feelings, offering her what she perceives as a closer connection to what she refers to as the universe, and reinforcing her conviction in the presence of God. Often, she expresses “Now, I see so clearly…”. Given the advanced stage of cancer, during the last months, she began preparing for her death, settling unfinished matters and expressing a deep interest in working on her legacy by writing legacy letters to leave for her daughters, so they can read them at different moments in their lives. She has been supporting her family through the process of anticipatory grief, striving to provide them with the opportunity to cultivate a sense of positive meaning and serenity regarding life after her passing. She considers herself an altruistic, compassionate person, leaving an earth spiritual mission and remains open to and accepting whatever the future may hold.

#### 3.3.2. Borderline Case

Some inconsistencies in the concept’s application are highlighted by the borderline example, which contains many but not all the concept’s defining attributes [30].

John, 44 years old, divorced, works as a philosophy teacher, and has a 12-year-old daughter. About six months ago, he was diagnosed with a plurimetastatic pancreatic tumor with poor survival time. John was referred to a specialized palliative care unit. At this time, he experienced a moment of profound hopelessness and suffering. He felt more depressed because he had a medical history of a previous refractory major depression. Therefore, he began pharmacological treatment with psilocybin (psychedelic), which he accepted. Simultaneously, he participated in art therapy sessions where he explored different expressive mediators, such as music and painting. After some time, he reported a unique mystic type-experience of enlightenment, which he described as his rebirth—both for himself and for others. Time no longer had a stopwatch, and his focus shifted away from himself, with a deeper focus on living in the present moment. However, when John tried to reconnect with his ex-wife to forgive her and live calmly and peacefully, she refused to visit him. Afterwards, he dealt with an intense fear of death that prevented him from achieving the expansion of consciousness and consequent existential integration.

#### 3.3.3. Contrary Case

It includes none of the main attributes of the concept [30]. Paul, a businessman of 57-year-old, with two children, described himself as a skeptical atheist. He has lived his entire life with a strong focus on businesses and his family’s well-being, providing everyone with a daily life that includes access to all material resources. He has lived with severe congestive heart failure for the past 5 years, and in recent months experienced a decline in his functional autonomy. He became unable to work and began struggling with basic activities of daily living. Clinically, he was given a poor prognosis. Since then, he has become socially withdrawn because he feels abandoned and doesn’t want to die. He adamantly refuses to resolve certain pending matters related to his business company, preventing his wife from taking over. He has been interacting with his healthcare professional and family more aggressively, not accepting any psycho-spiritual approach. He is willing to invest his savings in any place that offers a treatment claiming to cure him and does not accept his palliative condition.

### 3.4. Antecedents

According to Walker and Avant [30], antecedents are defined as events that arise before the occurrence of the concept. In the present concept analysis, two central antecedents were identified: (1) spiritual awareness and (2) the existential matrix.

**(1) Spiritual awareness**—results from an individual’s innate capacity (internal potential) and the opportunities provided by their surrounding context [48]. As death approaches, spiritual awareness tends to intensify [47], and altered states of consciousness pave the way for SA [41], which brings feelings of connection and fosters spiritual development and a reconfiguration of the self [36,41,45,49,54]. Individuals are presumed to possess and cultivate a natural capacity for spirituality, which may emerge more clearly in moments of vulnerability or deep reflection [49]. In an EoL context, physiological, psychological, and spiritual deterioration can foster connection with the transcendent and awaken awareness of human finitude [36,40,48]. As individuals face imminent death, spiritual awareness may intensify, triggering changes in worldview and beliefs [43,50].

**(2) Existential Matrix**—Existential crises can often shake an individual’s ontological grounding, affecting how they perceive and engage with their “storyworlds” [45]. Crises and a confrontation with unexpected and uncommon events can shatter personal routines and usual meaning-making. If something happens outside the canonical, we are eager to make sense of it. Narratives created about us and others can promote understanding or perpetuate harmful stereotypes and biases about spirituality. The development of interwoven personal and collective identities acknowledges that even as individuals shape their own distinct selves, they are still part of a wider societal narrative. In this way, each spiritual journey is influenced by multiple factors such as physical, emotional, cognitive, relational, and contextual conditions [43,47]. Therefore, SA stems from individual and collective experiences that shape the individual’s spiritual sensitivity [43,47], so that access to the person’s existential matrix occurs through biographical narratives. Naturally, these narrations are perpetually and inevitably evolving. Some are more enduring or impactful than others, but even these can be reformulated. Individuals navigate the world of narratives through stories they weave about themselves, others, and the world. The psychological turmoil inherent in life events—such as living with a life-limiting illness, losses and bereavement, and intense stress [36]—create fertile ground for changes in faith or spirituality and thus leads to SA. According to the findings, SA intensifies in moments of transpersonal crisis, when spiritual needs emerge and threaten a person’s identity [36,43,55,56]. Existential crises can lead to a redefinition of priorities [47], even in individuals without religious affiliation, leading them to connect with the sacred or a higher power. Such behavior can be interpreted as the manifestation of an innate psychological or neurobiological phenomenon, possibly associated with the search for meaning or integration in the face of approaching death [48], and which is only accessed through language that makes public the private experience of SA.

### 3.5. Consequences

Consequences are the events or incidents that may arise because of the occurrence of a concept, and which can often stimulate new ideas or avenues for research related to the concept [30]. SA allows for the revaluation of beliefs, the expansion of consciousness, and the acceptance of death as an essential part of life [36,45,50,53]. It simultaneously contributes to the control of existential suffering, thereby facilitating the attribution of meaning and purpose to existence [53]. In this sense, SA allows for the discovery of a personal purpose that fosters an identity and emotional transformation, supported by values of integrity, authenticity, and existential meaning [41,45]. Surrendering to a transcendent power fosters inner freedom and spiritual serenity, central elements for experiencing a “good death” [47,48,49,53]. In parallel, SA can contribute to the balance of physical (symptom control), social (presence of loved ones), emotional (sharing of emotions), and spiritual (inner peace) dimensions [53]. In the dying person, SA generates a sense of unity capable of stimulating wisdom, compassion, and altruism in strengthening bonds and relational qualities guided by values such as unconditional love, joy, and friendship [38,42,44,47]. These spiritual transformations lead to the strengthening of family ties, the desire to serve, the belief in immortality, and spiritual growth [41,50]. A desire to “leave a legacy” may emerge, evidenced by the symbolic continuity of one’s existence through future generations [42]. In parallel, people develop a better ability to cope with stress, accompanied by a decreased fear of death, levels of aggression, and attachment to material possessions [41].

### 3.6. Empirical Referents

The identification of empirical referents illustrates both the presence and practical relevance of the concept being analyzed [30]. Despite the scarcity of standardized instruments for assessing SA, some tools have been identified that can support professionals in their assessment, namely: (a) The Spirituality Scale, composed of 23 items, is a holistic instrument designed to assess beliefs, intuitions, lifestyles, practices, and rituals that reflect the spiritual dimension of the human being. In addition to supporting the implementation of spiritual interventions, this scale promotes a process of spiritual self-discovery, covering aspects such as spiritual relationships, a sense of sacredness, and awareness [49]; (b) The Non-dual Embodiment Thematic Inventory (NETI), composed of 20 items, was designed to evaluate qualities of the non-dual experience (states of self-transcendence) and SA [38]; (c) 30-Item Mystical Experience Questionnaire (MEQ30) was developed to measure the intensity of mystical experiences. It consists of four subscales: mystical (15 items); positive mood (6 items); transcendence of time and space (6 items); ineffability (3 items) [38]; (d) The Self-Transcendence Scale (STS) is composed by 15 items and “captures various aspects of self-transcendence, such as introspection, altruism, temporal integration, and transcendental awareness” [37]; and lastly (e) The 11 Dimensions Altered States of Consciousness (11D-ASC) is an instrument composed of 42 items divided into 11 subscales: “(1) Experience of Unity, (2) Spiritual Experience, (3) Blissful State, (4) Insightfulness, (5) Disembodiment, (6) Impaired Control and Cognition, (7) Anxiety, (8) Complex Imagery, (9) Elementary Imagery, (10) Audio-Visual Synesthesia, (11) Changed Meaning of Percepts” [38].

### 3.7. Definition of the Concept

**Figure 2:**
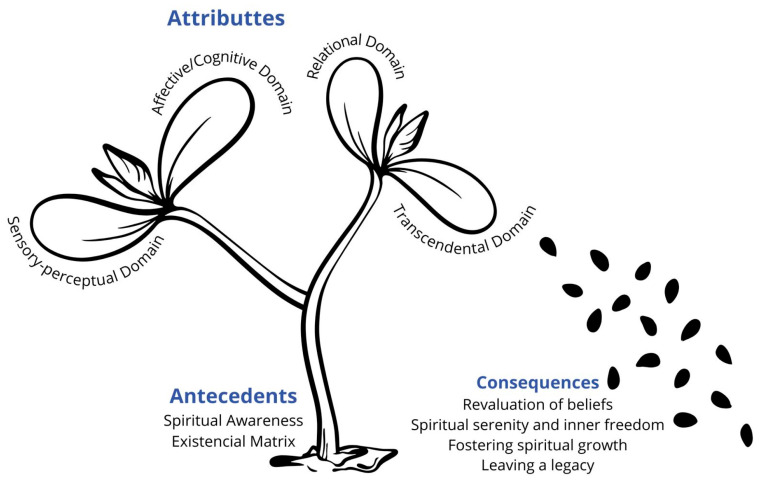
Representation of concept “Spiritual Awakening at the EoL”.

The operative meaning of the concept entails an interactive and reciprocal influence, wherein antecedents, attributes, and consequences inform and alter one another in an evolving process [30]. SA in EoL describes an inner transformational and subjective experience that involves intensifications of perception and a profound shift in one’s awareness of reality from an ordinary, finite sense of self to encompass a wider, infinite sense of truth or reality. This process of ego dissolution involves an individual’s deep connection with the meaning of existence towards the oneness experience with oneself, others, and the world. This idiosyncratic experience is focused on the present moment, can occur gradually or suddenly, spontaneously or induced, and allows one to find an expansive embodied way of being, of peace, gratitude, and unconditional love. For a better understanding of the concept, we used the flower bloom metaphor to represent the human journey at EoL toward SA. Figure 2 illustrates the suggested connection among the attributes that elucidate SA at the EoL, its antecedents, and the resultant consequences.

## 4. Discussion

This concept analysis sought to establish a definition of SA at the EoL, a construct that arises as a complex and interacting phenomenon throughout human experience. A comprehensive understanding and explanation of the concept is crucial for attaining excellence in palliative care assessment and delivery. The attributes of SA are evidenced in four domains—sensory–perceptual, affective-cognitive, relational, and transcendental—reflecting the phenomenological complexity of the experience. Changes in sensory-temporal perception arise in association with altered states of consciousness frequently documented in the neuroscientific and spiritual literature [38,39,40]. These changes support the idea that SA involves profound neuropsychological transformations that transcend everyday experience. In the affective-cognitive domain, the coexistence of positive and negative emotions and behaviors, observed at varying levels of consciousness [57], highlights the ambivalence inherent in human experience, even during processes of self-transcendence. Such complexity defies simplistic interpretations and suggests that SA is a multifaceted phenomenon that may include emotional and cognitive tensions that generate an expansion of moral, affective, and identity consciousness, which deepens the feeling of connection with humanity [57]. The relational dimension emphasizes empathy and social connection, the importance of human interactions in the context of SA, especially when mediated by contemplative practices and states induced by psychedelics [58,59]. The transcendental dimension, in turn, emphasizes the emergence of states of non-dual consciousness and experiences of unconditional love, which are central elements in the reorganization of the self and existential integration at the EoL [57,60,61,62]. The dissolution of the ego motivates an expanded spiritual awareness that leads to the dissolution of self-identity and the egoic sense of self, leaving the individual in a state of liberation from their challenges in life [40]. Moving from a pre-transition state of ego-based perception to a post-transition expanded consciousness can produce intense spiritual experiences [40], where unconditional love is a constitutive and fundamental force of self-awareness in the world and the bridge to the transcendent [60].

Through the antecedents of SA, including spiritual awareness and the individual’s existential matrix, the critical role of crisis and vulnerability events (proximity of death) in catalyzing processes of self-reconfiguration and expansion of consciousness is highlighted [38,63]. Regarding the consequences, the re-evaluation of beliefs, the expansion of consciousness, and the acceptance of finitude appear as outcomes of the SA process at the EoL [39,53]. The promotion of states such as inner serenity, compassion, and universal love contributes to spiritual well-being and the concept of a “good death” [36,60]. These findings support the premise that SA can have significant positive effects on the quality of palliative care, reinforcing the importance of emotional and spiritual support provided by healthcare professionals. Furthermore, studies indicate that such experiences occur regardless of formal religious affiliation, which highlights the universality of the phenomenon and its relevance in humanized and person-centered practices [38,40]. However, it is important to recognize that, although positive effects predominate, experiences of discomfort may arise, requiring careful and personalized assessments. This discussion highlights the need for future research that integrates neuroscientific, psychological, and spiritual approaches to deepen the understanding of SA in diverse cultural and religious contexts, contributing to the development of therapeutic strategies that value the existential and spiritual dimension of human beings in EoL processes.

### 4.1. Study Limitations

This study has some limitations that should be considered when interpreting the findings. First, SA is a complex concept and, given its subjective nature and variability across cultures and religious traditions, a clear and consensual conceptual definition is challenging. Regarding the literature review, there was a limitation in the sources consulted, which excluded databases from fields such as philosophy, theology, and religious sciences, which could offer distinct conceptual approaches. Moreover, as spiritual awakenings exist beyond conventional medical and scientific paradigms and have consequently undergone limited investigation, they are hardly addressed in media and public discourse. Consequently, individuals possessing this type of experience typically lack a framework to comprehend, assimilate, and integrate the experience for optimal benefit. Other study limitations include language restriction bias, publication bias, risk of bias assessment and exclusion of theses/dissertations, which may compromise the validity of the results.

While literature on phenomena of spiritual emergence and transpersonal crisis has distinguished conventional mental illness from spiritual development [64,65], more evidence on ego-identity crisis lived in EoL is required. Furthermore, due to the ambiguity surrounding whether such experiences are healthy or pathological, the capacity of healthcare and scientific institutions to respond effectively is constrained. Lastly, mixed-method studies are needed that, through self-report questionnaires, allow for a quantitative assessment of its intensity and respective attributes and explore people’s lived experience regarding the phenomenology and transformative effects of SAs in EoL care.

### 4.2. Implications for Practice

Promoting a good death is one of the goals of palliative care, which aims to ensure adequate symptom control, respect for human dignity, and psychospiritual support during death [53,66]. It is essential that healthcare teams broaden their perspective on EoL care, integrating spirituality and being open to integrative assessment approaches aimed at more holistic and person-centered care [67]. In this vein, approaching SA has implications for all healthcare areas where existential questions and crises may arise. It also has consequences for the person or team that provides spiritual care, which should be competent in their provision. In this sense, professionals must acknowledge spiritual care as a systematic and deliberate process. This encompasses the identification of spiritual needs and resources, comprehension of the patient’s unique context, formulation of a personalized spiritual care plan, engagement of relevant healthcare and spiritual care professionals, provision of care, and assessment of its outcomes [27,68]. This process is grounded in the acknowledgment of each individual’s ontological foundation and interpreted through the “meaning-making matrix,” which underscores the necessity for professionals to perceive the individual receiving care as a narrative entity, necessitating personalized and relational care that is continuously evaluated and adjusted [27,68].

To deal with SA experiences in healthcare settings, a multifaceted approach is needed. This includes recognizing spirituality as a determinant of health, integrating spiritual care into routine practice, and providing education and training on spiritual care competence for healthcare professionals [69,70]. Furthermore, it is necessary to implement relational strategies, such as compassionate presence and active listening, to alleviate existential suffering, and incorporate spiritual practices, such as contemplative practices and legacy-based interventions [71,72], to enhance patients’ spiritual well-being in palliative care. While spiritual care competence is not an innate skill, it requires spiritual sensitivity, the capacity for intersubjective connection, and adequate professional training, which translates into spiritual growth and well-being for both caregivers and those receiving care [27,73,74,75]. Healthcare institutions should promote training and ongoing education for professionals on the use of narrative/storytelling and validated decision-making support tools as a means of identifying patients’ spiritual needs and assessing the impact of spiritual care [24,66,76,77,78].

## 5. Conclusions

The concept of SA at the EoL reveals itself to be a complex and multifactorial phenomenon, with a profound impact on a person’s confrontation with finitude. Recognizing and integrating SA into palliative care allows for a more comprehensive understanding of human consciousness. This concept analysis revealed four attribute domains: (1) sensory–perceptual domain; (2) affective/cognitive domain; (3) relational domain; and (4) transcendental domain. Moreover, spiritual consciousness and the existential matrix were antecedents to this concept; revaluation of beliefs, finding spiritual serenity and inner freedom, fostering spiritual growth, and the desire to leave a legacy were its consequences. Transformation seems to be a notable consequence of SA experiences. That spiritual transformation represents a modification in the meaning system that underpins an individual’s self-definition, life interpretation, overarching purposes, ultimate concerns, values, meanings, and associated life orientations. It is necessary to invest in more studies with a transdisciplinary approach to philosophy, psychology, nursing and other care professions to establish clinical practices that are more congruently humanizing and sensitive to the spiritual dimension of the human being. Moreover, comprehending the phenomenology of SA experiences enhances multicultural competence and mitigates the likelihood of professionals pathologizing individuals’ interpretations of sacred, transpersonal, and mystical occurrences.

## Figures and Tables

**Table 1:** Concept-defining attributes.

Domain	Attributes
Sensory–perceptual Domain	Changes in temporal–spatial perceptions [36,37]
	Intense body sensations [38]
	Internal transformative energetic experiences [38,39]
Affective/cognitive Domain	Sense of morality [38,40,41]
	Awareness of the present moment [38,40,41]
	Expanded consciousness [38,40,41,42]
	Universal outlook [38,40,41]
	Positive affect [43,44,45]
	Here and now experience [45,46]
	Unifying silence [36,42,43,44,45,46]
	Feeling of connection with humanity [36,38,40,46,47]
Relational Domain	Dissolution of bonds [36,43,45,47]
	Psychological and social detachment [36,43,45,47]
	Nonmaterialism [36,43,45,47]
	Altruism, compassion, empathy and forgiveness development [36,43,45,47]
	Desire to give and receive love [38,43,45,48,49]
	Conscious distancing from formal religious institutions [38]
Transcendental Domain	Fusion with a supreme reality [38,39,45]
	Deep existential integration [36,38,39,40]
	Finding meaning [48,50]
	Desire to leave legacy [42]
	Search for inner peace [36,47,51,52]
	Sense of belonging to a higher and transcendental reality [42,51]

## Data Availability

All data generated or analyzed during this study are included in this article. This article is based on the first author’s Master’s dissertation in Palliative Care at the School of Health Sciences—Polytechnic University of Leiria (supervised by Carlos Laranjeira).

## Supplementary Materials

The following supporting information can be downloaded at: https://www.mdpi.com/article/10.3390/nursrep15100358/s1, Table S1: Characteristics of the studies included in the review (N = 21).

## Public Involvement Statement

No public involvement in any aspect of this research.

## Guidelines and Standards Statement

This manuscript was drafted in accordance with the stages proposed by Braun and Clarke [30].

## Abbreviations

The following abbreviations are used in this manuscript:

CINAHL	Cumulative Index to Nursing and Allied Health Literature
EAPC	European Association of Palliative Care
EoL	End-of-Life
MeSH	Medical Subject Headings
NGO	Non-Governmental Organization
PC	Palliative Care
SA	Spiritual Awakening
