# Supplementary material for: Seeking Something Beyond Themselves: A Concept Analysis of Spiritual Awakening Experiences at the End of Life
Source: Nurs Rep. 2025 Oct 8;15(10):358. doi: 10.3390/nursrep15100358 (PMC12567353; doi:10.3390/nursrep15100358)
Supplement: Supplementary file 1 [file nursrep-15-00358-s001.zip › nursrep-3874270-supplementary.pdf]

Supplementary Table S1. Characteristics of the studies included in the review (n = 21)

#	Author(s)/ Year / Country	Type of Study/Paper	Aims	Setting/ Participants	Key findings
36.	(Taylor, 2018)<br><br>United Kingdom	Qualitative study	The aim was to develop a typology of sudden spiritual awakening experiences by identifying and characterizing the major forms or modes in which they occur, and to explore potential psychological and energetic mechanisms underlying these experiences.	19 self-reported cases of sudden spiritual awakening, drawn from a larger sample that also included gradual awakening cases.<br>Data were collected retrospectively from participants' self-reports.	Sudden spiritual awakenings occur in two main forms: ego-dissolution, involving a structural collapse of the self, and kundalini-like, involving an explosive release of energy. Both types are often triggered by intense psychological turmoil such as bereavement, depression, or stress. The study suggests these experiences reflect the release of energy normally tied to specific psychological or bodily functions, explaining their distinct characteristics.
37.	(Abu Khait et al., 2024)<br><br>USA	Quantitative study	This study examined how self-transcendence mediates the relationship between reminiscence and death anxiety in Jordanian older adults, suggesting that psychiatric nurses can enhance reminiscence therapy by fostering self-transcendence to reduce death anxiety.	A total of 319 older adults from Jordan participated in the study, answering a survey.	"Bitterness Revival", gender, life-threatening illness, psychiatric history, and work sector significantly predicted death anxiety. Reminiscence functions 1, 2, and 5 predicted self-transcendence. Self-transcendence partially mediated the relationship between "Bitterness Revival" and death anxiety. This knowledge has practical implications for psychiatric nurses about the importance of developing reminiscence interventions to promote self-transcendence and ease death anxiety.
38.	(Corneille and Luke, 2021)<br><br>Brazil	Quantitative study	The objectives of this study were to investigate the phenomenological characteristics of Spontaneous Spiritual Awakenings (SSAs) and Spontaneous Kundalini Awakenings (SKAs), compare them with other altered states of consciousness (including psychedelic experiences), and examine their impact on well-being, as well as the role of personality trait absorption and	152 self-selected participants completed online questionnaires about their SSA/SKA experiences.	SSA/SKAs were found to be powerful, mostly positive transformative experiences, phenomenologically similar to psychedelic states, with personality and neurological traits predicting their occurrence.

			temporal lobe lability as predictors of these experiences.		
39.	(Taylor, 2019)<br><br>United Kingdom	Qualitative study	The aim was to investigate and clarify the nature of awakening experiences, specifically focusing on their occurrence outside religious and spiritual traditions or practices.	161 individuals provided a single written account of a personal awakening experience. These participants were recruited from three main settings: adult students attending Positive Psychology courses taught by Taylor at the University of Manchester; individuals responding to an open invitation on Taylor's website to share awakening experiences; and participants at his workshops.	Awakening experiences occur spontaneously, outside religious contexts. A psychological approach is proposed, together with the use of a more neutral term to describe these experiences.
40.	(Renz et al., 2017)<br><br>Switzerland	Mixed method study	To investigate the interrelationships between fear, pain, denial, and spiritual experiences in terminally ill patients, and to explore how these factors relate to transformations in perception and consciousness as patients approach death.	The study observed a sample of 80 dying cancer patients in palliative care units at two Swiss cantonal hospitals.	Most patients experienced fear and pain at least once, while many also had spiritual experiences and partial transformations of perception, only partly influenced by medication. There are associations between fear, pain, denial, spiritual experiences, and perceptual transformation. No patient experienced uninterrupted distress, and many appeared to die peacefully. Prior near-death or spiritual experiences may ease the dying process.
41.	(Woollacott et al., 2021)<br><br>India	Qualitative study	The aim of the study was to explore the phenomenology, energetic characteristics, and subsequent behavioral and physiological transformative effects of spiritually transformative experiences (STEs), particularly those with an energetic component such as kundalini awakenings.	342 individuals who completed a subset of questions from the Kundalini Experiences Inventory. Data were collected via self-administered questionnaires using open-ended questions from the Kundalini Experiences Inventory.	Energetic spiritual awakenings were described as mystical experiences involving expansion, out-of-body awareness, and unusual energy flows. They were commonly triggered by spiritual focus, guidance from spiritually advanced individuals, or intense meditation/prayer. Transformational effects included heightened sensitivity, creativity, and shifts toward unity, service, and spiritual awareness. Many participants reported that healthcare professionals lacked understanding of these experiences, limiting support.
42.	(Asgeirsdottir et al., 2013)<br><br>Iceland	Qualitative study	The aim of this study was to explore spirituality from the perspective of individuals receiving palliative care and to	Qualitative data interviews with ten patients (five women and five men) with cancer treated in the	Thematic analysis revealed that participants regarded spirituality as a vital dimension linked to meaning, purpose, and transcendence. Both religious and non-religious expressions emerged, including the role of family, belief in

			examine their experiences of spirituality and its impact on their lives and well-being.	Palliative Care Unit of the National University Hospital of Iceland.	God or a higher power, and spiritual practices that fostered strength, inner resources, and hope. Nine participants explicitly described their spirituality as faith.
43.	(Tanzi et al., 2023)<br><br>Italy	Qualitative study	The aim was to explore the perceptions about the spirituality of people with life-limiting illnesses.	Twenty-one patients suffering from rheumatic, hematologic, neurodegenerative and respiratory chronic diseases.	Participants were frequently unable to provide a clear definition of "spirituality". The data revealed four overarching themes: the definition of spirituality, internal dialogue, the expression of spirituality in everyday life, and the process of taking stock. Religion did not emerge as a response to spiritual suffering.
44.	(Bozkurt and Yildirim, 2024)<br><br>Turkey	Quantitative study	The aim was to investigate the impact of individualized reminiscence therapy on global distress, physical and psychological symptoms, life satisfaction, and self-transcendence among palliative care patients.	The study was conducted in a single-center palliative care service in western Turkey and included 48 patients without cognitive impairment who were able to communicate. Of these, 44 patients completed the study. Eligible participants were randomly assigned to one of three groups prior to the intervention: reminiscence therapy (intervention), unstructured social interviewing (placebo), or control, with 16 patients allocated to each group.	No significant changes were observed in physical or total symptom burden ($p > 0.05$). Both intervention and placebo groups showed reductions in general distress and psychological symptoms ($p < 0.05$), though differences between groups were not significant ($p > 0.05$). Significant group × time interactions were found for life satisfaction and self-transcendence ($p < 0.001$), with the intervention group showing the greatest improvement.
45.	(Maurya et al., 2023)<br><br>United States	Qualitative study	The purpose of the present study was to explore and develop a grounded theory of how spiritual awakening is conceptualized and experienced, capturing its process and evolution across diverse spiritual traditions.	34 eminent spiritual teachers from diverse cultural backgrounds (American, Asian, European, Mexican) and spiritual traditions (e.g., Buddhism, Yoga, Hinduism, Taoism, Christianity, mindfulness practices). Interviews were conducted (remotely or in person, though setting details are not specified) to gather in-depth insights into their spiritual experiences and teachings.	Analysis of the interviews resulted in the development of the Spiritual Awakening Evolution (SAE) model, which outlines four phases of spiritual awakening: an initial transient shift in perception, further practice to support spiritual growth, surrendering, and an ongoing journey of development. Given the transcultural nature of these experiences, the SAE model can help counselors understand clients' spiritual needs and the significance of their awakening, with implications for practice, training, and future research.

46.	(Kilrea, 2018)<br><br>Canada	Discussion paper	This study explored and advanced the psychological understanding of the phenomenon of awakening, which includes both spiritual and secular experiences.	NA	The article concludes that awakening is a real, measurable phenomenon associated with profound psychological benefits, but its exact nature, scope, and mechanisms require further rigorous investigation and clearer conceptualization.
47.	(Mok et al., 2010)<br><br>China	Qualitative study	The aim was to explore the experiences and perceptions of spirituality and spiritual care among terminally ill Chinese patients.	Phenomenological interviews were conducted in 2007 with a convenience sample of fifteen terminally ill Chinese patients.	Participants perceived spirituality as an abstract, personal belief that provides strength and relates to life's meaning. It was viewed as a multidimensional integration of body and mind, with acceptance of death and 'letting go' fostering serenity. Key themes included deriving meaning through relationships, self-reflection on responsibilities, and fulfillment of obligations. Inner spiritual well-being was linked to faith and understanding life's possibilities, including after death. While participants did not expect nurses to provide spiritual care, high-quality interpersonal care was experienced as spiritually supportive.
48.	(Collin, 2012)<br><br>United Kingdom	Qualitative study	The aim of this study was to explore the triggers, experiences, and ambivalent feelings underlying terminally ill patients' search for connection with a higher power in a palliative care setting.	A purposive sample of six adults from a Scottish hospice, two women and four men (ages ranged from 47 to 73 years), in various stages of terminal illness, who were receiving home-based palliative care from clinical nurse specialists (CNSs).	The results underscore the ambivalence patients expressed toward a transcendent being, perceived both as a source of anger and blame and as a source of comfort and hope for an afterlife. The study also indicated that health professionals may feel constrained in facilitating such discussions, seeking to protect both patients and themselves from engaging with an unfamiliar and complex domain.
49.	(McClintock et al., 2016)<br><br>United States	Quantitative study	To identify potential universal dimensions of spirituality across cultures using large-scale, multi-national data, and to examine how these dimensions are associated with common psychiatric conditions.	From 2014 to February 2015, 5,512 participants (41% female; mean age = 29 years, age range: 18–75 years) were recruited from the crowdsourcing websites Zhubajie.com and Mturk.com among residents of China (N = 3,150), India (N = 863), and the United States (N = 1,499).	Five universal dimensions of spirituality—love, interconnectedness, altruism, contemplative practice, and religious/spiritual reflection—were identified across China, India, and the U.S. Love, interconnectedness, and altruism consistently protected against psychiatric symptoms, while the effects of contemplative practice and religious/spiritual reflection varied by country. Overall, these findings suggest that distinct aspects of spirituality may offer cross-cultural mental health benefits.
50.	Long and Woollacott,	Mixed method study	The study aimed to deepen understanding of the	A total of 834 questionnaires collected through the Near Death	The findings indicate that participants who experienced NDEs showed significant transformations in values and

	2024)<br><br>USA		phenomenon of long-term transformational effects of near-death experiences (NDEs).	Research Foundation in the USA were analyzed and compared with responses from 42 individuals who had experienced life-threatening events (LTEs) but did not report near-death experiences.	spiritual attitudes compared to those facing life-threatening events without NDEs. These changes included stronger belief in the divine and the afterlife, reduced fear of death, greater compassion, and a heightened sense of life's meaning, with a notable shift in priorities toward spiritual or religious aspects of life.
51.	(Arnold and Lloyd, 2014)<br><br>Canada	Mixed method study	The aim was to investigate the prevalence and thematic properties of complex transcendence experiences among terminally ill patients, using multimodal methods to analyze the metaphors they employ to describe these experiences and their potential role in enhancing communication in palliative care.	The final sample consisted of 85 patients reports. Eligible patients were English-speaking adults treated at the San Diego Hospice and Institute for Palliative Medicine (SDHIPM) between 2009 and 2011.	Terminally ill patients may experience unexpected and profound cognitive shifts—transcendence experiences—that can significantly change their self-perception and eliminate fear of death. Using a multimodal methodology, the study identified complex emergent metaphors that patients use to describe these experiences. The findings suggest that understanding these complex metaphors has considerable potential to improve communication and care in palliative settings, beyond the insights gained from conventional end-of-life metaphors.
52.	(Moestrup and Hvidt, 2016)<br><br>Denmark	Qualitative study	This study aims, through semi-structured interviews with Danish hospice patients and participant observation, to elucidate how patients reflect on and engage with their potential faith.	Seventeen Danish palliative care patients (eleven women, six men, mean age 62 years) participated in the study. The interviews, which included open-ended questions, lasted an average of 40 minutes and took place in the patients' rooms.	The results revealed three dimensions of faith: (1) knowing (cognition), (2) doing (praxis), and (3) being (meaning). The study concludes that most patients possessed a tentative yet meaningful faith, which, while important in their situation, was neither well understood nor frequently discussed or practiced.
53.	(Bovero et al., 2020)<br><br>Italy	Qualitative study	To explore how family caregivers (FCs) and health-care providers (HCPs) perceive the concept of a good death, their attitudes toward death, and feelings of interconnectedness, and to examine how these attitudes and feelings are associated with the importance they attribute to features of a good death.	The study included 49 participants directly involved in the care of terminally ill cancer patients: 24 family caregivers (49%) and 25 health-care providers (51%) from "Citt'a della Salute e della Scienza" Hospital and the "V. Valletta" Hospice in Turin.	Participants emphasized physical, social, emotional, and spiritual features as essential to a good death. Greater importance placed on patient awareness, acceptance, and inner peace was associated with lower death avoidance, higher acceptance of death, and stronger feelings of interconnectedness.

54.	(Bonavita et al., 2017)<br><br>USA	Qualitative study	The aim was to examine the perceptions of interfaith spiritual care received through a voluntary palliative care organization.	Ten adults (4 men and 6 women) aged 45 to 90 were selected from a USA voluntary palliative care organization.	Four main themes emerged: the vital role of spirituality in end-of-life care, definitions and boundaries of spirituality and interfaith spiritual care, unique aspects of interfaith spiritual care, and unmet spiritual needs. Findings highlight the importance of spirituality in hospice care and suggest ways to better address individual spiritual experiences
55.	(Williams, 2012)<br><br>USA	Qualitative study	To explore the meaning of self-transcendence — the ability to go beyond the self — among patients who have undergone stem cell transplantation.	Eight participants (4 men and 4 women), aged 45–63, who had undergone stem cell transplantation within the previous year, were recruited from a major academic oncology center in the USA.	Self-transcendence emerged as a process triggered by the participants' suffering from treatment, confrontation with mortality, and the inner strength they drew from spiritually influenced turning points. Human connections alleviated feelings of vulnerability, and participants described transformative changes both physically and personally during recovery.
56.	(Kukla et al., 2022)<br><br>Germany	Qualitative study	The aim was to explore how individuals cope with the awareness of their own mortality (due to age or life-limiting illness), to understand their strategies and wishes, and to use these insights to inform the development of supportive concepts that enhance psychosocial well-being and reduce existential distress.	Twenty-one semi-structured interviews were conducted with people over 80 years of age (n = 11) and with some limiting disease (n = 10). The interviews were conducted privately, free from distractions, at each participant's preferred location: at home (n = 16), in a hospice (n = 3), in a care home (n = 2), or on the premises of the University (n = 1).	No major differences emerged between participants aged 80+ and those with life-limiting diseases regarding coping strategies or attitudes toward the end of life. Both groups emphasized that theoretical education, practical preparations (e.g., funerals), open conversations, personal reflection, and spiritual contemplation fostered assurance, self-determination, and relief. Participants further highlighted the need for confrontation as well as low-threshold, accessible, and flexible services to address their existential and spiritual needs.

NA: Not applicable
